# Transcriptome analysis of rats with chronic unpredictable mild stress treated with electroacupuncture

**DOI:** 10.1002/brb3.70045

**Published:** 2024-09-18

**Authors:** Xiaoli Chang, Ying Wang, Yi Hou, Weilu Cheng, Shaozong Chen

**Affiliations:** ^1^ Acupuncture Research Institute Shandong University of Traditional Chinese Medicine Jinan China; ^2^ School of Acupuncture and Tuina Shandong University of Traditional Chinese Medicine Jinan China

**Keywords:** depression, electroacupuncture, transcriptome

## Abstract

**Background:**

Depression remains one of the most prevalent psychiatric disorders, with many patients not responding adequately to available treatments. Electroacupuncture (EA), a nonpharmacologic therapy, holds great promise for alleviating depressive symptoms. In this study, RNA sequencing (RNA‐Seq) was used to identify genome‐wide alterations in the hippocampus of rats after chronic unpredictable mild stress (CUMS) and EA treatments to further elucidate the mechanism by which EA ameliorates depression to provide a basis for the clinical application of EA in stress‐related diseases.

**Methods:**

The sucrose preference test (SPT), open field test (OFT), and forced swimming test (FST) were used to investigate the ability of EA at *Baihui* (GV20) and *Taichong* acupoints (LR3) to improve depression‐like behavior in rats subjected to CUMS. Subsequently, RNA‐Seq analysis revealed transcriptomic profiles of the hippocampus of rats subjected to CUMS in which EA ameliorated depressive behavior. Finally, the expression profiles of major differentially expressed genes were tested by real‐time quantitative polymerase chain reaction (qRT‐PCR) to determine the accuracy of the RNA‐Seq results.

**Results:**

Rats subjected to CUMS exhibited depressive‐like behaviors, such as decreased sucrose consumption in the SPT (*p *< .001), decreased time in the central area of the OFT (*p *< .001), and increased immobility in the FST (*p *< .01). Importantly, rats subjected to CUMS and treated with EA showed increased sucrose consumption (*p *< .001), increased time spent in the central area of the OFT (*p *< .001) and decreased immobility in the FST (*p *< .01). Sixty‐three genes that were differentially expressed following CUMS were altered by EA; most of these were associated with immune pathways. Compared with those in the control group, the expression levels of Colla2 (*p *< .001), Col3a1 (*p *< .001), Psmb9 (*p *< .01), and Tap1 (*p *< .01) in the hippocampus of rats subjected to CUMS were lower. The changes in the expression of these genes were reversed by EA treatment.

**Conclusion:**

EA at GV20 and LR3 attenuated CUMS‐induced depression‐like behaviors by regulating the expression of specific genes such as Colla2, Col3a1, Psmb9, and Tap1.

## Graphical abstract

EA at GV20 and LR3 ameliorated depression‐like behavioral deficits in CUMS rats. EA modulates depression by regulating the expression of particular genes such as Colla2, Col3a1, Psmb9 and Tap1.

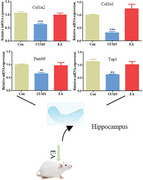

## 1 INTRODUCTION

Depression is a common disease characterized by mood disorders with the main symptom being a marked and persistent low mood. According to the Global Burden of Disease Research Report, depression is the main cause of disability (Otte et al., 2016). The outbreak of the novel coronavirus disease in 2019 has brought great changes to people's lives around the world, and psychological pressure continues to increase. An article published in the Lancet showed that after the start of the COVID‐19 epidemic in 2020, the number of patients with depression in the world increased by 27.6%, and the number of patients with anxiety increased by 25.6% (Santomauro, 2021). Drugs are currently the main means of antidepressant treatment. However, Western medicine approaches for treating depression are limited in clinical practice due to side effects such as gastrointestinal reactions, sleepiness, and suicidal tendencies (Kverno & Mangano, 2021).

Acupuncture or electroacupuncture (EA) is a promising nonpharmacological treatment for improving depression that can be used as an alternative to pharmacotherapy or as an adjunctive therapy to improve outcomes (Smith et al., 2018). This was demonstrated in a multicenter, randomized, positive‐controlled clinical trial in which 242 perimenopausal women with mild to moderate depressive symptoms were randomized to receive 36 sessions of EA or escitalopram. The study revealed no differences in depressive scores, menopause specific quality of life, or serum sex hormones between the two groups. But in the follow‐up, EA has a nice long‐term antidepression effect (S. Li et al., 2018). In addition, compared with pharmacological treatments, acupuncture has the advantages of low cost and minimal side effects (Yang et al., 2022). The main causes of depression are brain inflammation and disruption of the blood–brain barrier, which leads to neuronal loss in brain regions (especially the hippocampus), decreased monoamine levels, and impaired brain network connectivity. Research on animal models and patients with depression has shown that acupuncture can increase the plasticity of brain regions and networks, reduce brain inflammation, and possibly alleviate depression (X. Wang et al., 2016; Yang et al., 2021). There is strong evidence that abnormal gene expression makes it difficult to cure conditions such as depression (Gonzales et al., 2023; Park et al., 2019). Recent studies have reported that abnormal gene expression in the prefrontal cortex and hypothalamus of depressed rats tends to be reversed after EA treatment (Y. Wang et al., 2024; Zheng et al., 2019). However, the mechanism through which acupuncture affects depression remains poorly understood.

The chronic unpredictable mild stress (CUMS) model is among the most common and frequently used models of depression. The model involves exposing the animal to a series of minor‐intensity stressors at unpredictable times over several weeks (Willner et al., 1987). CUMS leads to the disruption of homeostasis, causing somatic, physiological, neurobiological, biochemical, and behavioral disturbances, which are similar to symptoms in patients with depression (Chen et al., 2023; E. Li et al., 2023). Therefore, in this study, we utilized the CUMS rat model to investigate the effects and mechanisms of EA on depression. Apoptosis, atrophy, and synaptic loss of neurons in the hippocampus, a major brain region that regulates emotions, are directly associated with the development of depression (Tartt et al., 2022; Wu & Zhang, 2023). EA attenuates depression‐like behaviors in rats by modulating oxidative stress, neuroinflammation, and neuroplasticity in the hippocampus (Han et al., 2018; Jiang et al., 2024; Shen et al., 2024). In addition, a large number of genes, such as the monoamine‐related gene polymorphisms and oxytocin receptor gene polymorphism, have been found to play a salient role in structural alterations of the hippocampus in patients with depression (Na et al., 2018; Phillips et al., 2015). Given the important role of the hippocampus in the pathogenesis of depression and EA antidepressants, RNA sequencing (RNA‐Seq) was used to determine genomic changes in the hippocampus of rats following CUMS and EA treatment. Our study provides new mechanistic insights into the antidepressant effects of EA in rats subjected to CUMS.

## 2 MATERIALS AND METHODS

### 2.1 Animals and groups

**FIGURE 1:**
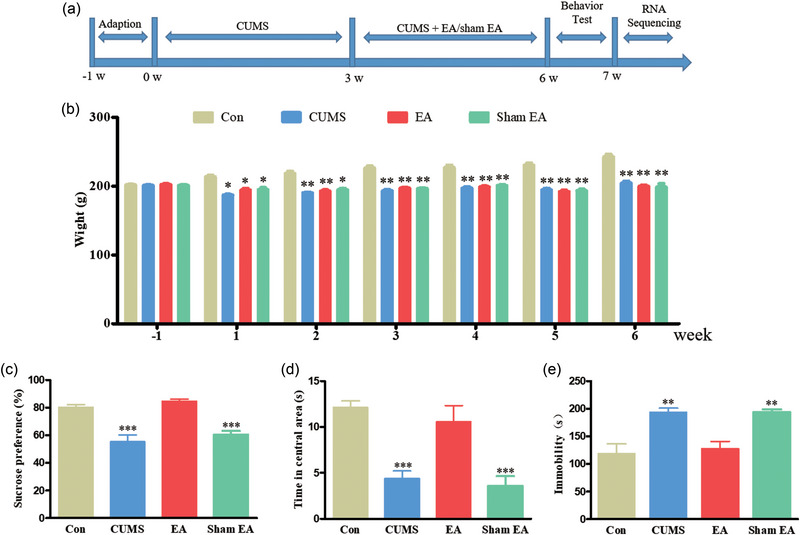
Electroacupuncture (EA) ameliorated depression‐like behavioral deficits in rats subjected to chronic unpredictable mild stress (CUMS). (a) The experimental schedule of CUMS, EA, or Sham‐EA treatment, behavioral tests, and RNA sequencing. (b) Effect of EA on body weight in a rat depression model. (c–e) EA at GV20 and LR3 in rats subjected to CUMS significantly elevated the sucrose preference rate and residence time in the central area and reduced immobility time. The data are expressed as the means ± SD; *n* = 8–10; ^*^ *p *< .05, ^**^ *p *< .01, ^***^ *p *< .001, compared with the control group (Con group).

Eight‐week‐old male Wistar rats weighing 200–210 g were purchased from Jinan Pengyue Laboratory Animal Co., Ltd. Rats were raised in groups of three per cage with free access to food and water under a 12 h light/dark cycle in a suitable environment for temperature (20–25°C) and humidity (40%–50%). All procedures were conducted in accordance with the National Institutes of Health Guidelines for the Care and Use of Laboratory Animals and approved by the Institutional Ethics Committee of Shandong University of Traditional Chinese Medicine (SDUTCM20230221002). The rats were acclimatized to their new environment for 1 week, and the body weight during the first week was used as a reference point. According to the random numbers table, the rats were randomly divided into four groups (*n* = 12 per group): the control group (Con group), CUMS group, EA group, and Sham EA group. The CUMS method was used to establish a rat model of depression, except in the control group. After 3 weeks of exposure to CUMS, rats in the EA group and Sham EA group were treated with EA or Sham EA continuously for 3 weeks, respectively. Subsequently, all rats were used for behavioral testing, and four rats in the Con group, CUMS group, and EA group were used for RNA‐Seq. The detailed experimental scheme is shown in Figure 1a.

### 2.2 CUMS procedure

Rats in the Con group were kept under normal feeding conditions without any processing and had free access to food and water. The other three groups of rats were exposed to CUMS for 6 weeks. The CUMS procedure was performed as previously described with a slight modification (Y. Li et al., 2022). The different stressors the rats were exposed to are shown in Table 1. The rats were randomly exposed to different stressors every day, making it impossible for the animals to predict the stimulus. The same stressor was used on consecutive days.

**TABLE 1:** Summary of the program for chronic unpredictable mild stress (CUMS) stimulus.

Stressors	Illustration
Cage tilting	Cage tilting at 45° for 24 h
Swimming in cold water	3 min; 0°C
Water deprivation	Water deprivation for 24 h
Food deprivation	Food deprivation for 24 h
Horizontal shaking	Horizontal shaking for 15 min
Tail biting	Tail biting for 1 min
Heat stress	45°C heat stress for 5 min
Alternating light/dark cycles	Alternating light/dark cycles for 24 h
Wet bedding	Wet bedding for 24 h
Restraint	Restrict in a small device for 2 h

### 2.3 EA stimulation

Rats in the EA group were treated with EA for 21 consecutive days. After the rats were anesthetized with 5% isoflurane (3.5%; RWD), they were moved to the EA operating platform with a 2% isoflurane concentration. Stainless steel needles (0.25 mm in diameter; 25 mm in length; Suzhou Medical Appliance Factory) were inserted into *Baihui* (GV20) and *Taichong* (LR3) to a depth of approximately 0.5 mm. A Huatuo Acupoint Neurostimulator (SDZ‐V; Huatuo Medical Technology Co., Ltd.) was connected and an electric current was applied to the needles. EA was carried out at a frequency of 2 Hz and an intensity of 0.1–3 mA once a day for 20 min. The Sham EA group did not receive electrical stimulation, and the needles were attached to the surface of GV20 and LR3 so that the needles were in contact with the skin but not inserted into the acupoints. Rats in the Con group and CUMS group were subjected to anesthesia only.

### 2.4 Behavioral tests

#### 2.4.1 Body weight measurement

During this period, the body weights of the rats in each group were measured at 9 a.m. each week using a weighing scale.

#### 2.4.2 Sucrose preference test

Rats were deprived of water and food for 12 h before the preference test. During the preference test, the rats were housed in individual cages and then given free access to two bottles containing sucrose solution (1% sucrose, 200 mL) and water (200 mL) for 12 h. During this 12‐h period, the positions of the two bottles were changed so as not to interfere with their drinking habits. The consumption of the sucrose solution and tap water was calculated by weighing the bottle. The sucrose preference value was calculated as follows: sucrose consumption (g) × 100%/[sucrose consumption (g) + water consumption (g)].

#### 2.4.3 Open field test

Rats were placed in the open area in the center of the apparatus (100 cm × 100 cm × 40 cm) and allowed to explore freely for 5 min. The time the rats spent in the center area was recorded using SuperMaze V2.0 (Shanghai Xin‐ruan Information Technology Co.). After each rat was tested, the site was wiped with a damp cloth and blown dry with a hot air blower.

#### 2.4.4 Forced swimming test

Rats were placed one by one in a glass cylinders (21 cm in diameter; 46 cm in length) filled with water (23–25°C) to a depth of 30 cm so that their hind limbs could not touch the bottom of the cylinder. The water was exchanged between the rats. Six‐minute tests were recorded using SuperMaze V2.0 (Shanghai Xin‐ruan Information Technology Co.), and the immobility time was measured.

### 2.5 RNA extraction

Considering that Sham EA treatment did not improve depressive‐like behaviors in the rats subjected to CUMS, sequencing analysis was performed on the hippocampus of rats in the Con group, CUMS group, and EA group. Rats were sacrificed under deep anesthesia. The hippocampus of each rat was extracted after perfusion with phosphate‐buffered saline, immediately frozen on dry ice, and stored at −80°C. Total RNA was extracted from the tissue using TRIzol Reagent according to the manufacturer's instructions and then analyzed using a 5300 Bioanalyzer (Agilent Technologies Inc.). Only high‐quality RNA samples (OD 260/280 = 1.8–2.2, OD 260/230 ≥ 2.0, RIN ≥ 6.5, 28S:18S ≥ 1.0, > 1 µg) were used for construction of the sequencing library.

### 2.6 Library preparation and sequencing

RNA purification, reverse transcription, library construction, and sequencing were performed by Shanghai Majorbio Biopharm Biotechnology Co., Ltd. according to the manufacturer's instructions. The hippocampal RNA‐Seq libraries were prepared according to the Illumina Stranded mRNA Prep, Ligation from Illumina using 1 µg of total RNA. Messenger RNA was first isolated by polyA selection using oligo (dT) beads and then fragmented using fragmentation buffer. Bidirectional cDNA was then synthesized using a SuperScript bidirectional cDNA synthesis kit (Invitrogen) and random hexamer primers (Illumina). The synthesized cDNA was then subjected to end repair, phosphorylation, and A‐base insertion according to Illumina's library construction protocol. The 300 bp cDNA target fragment size was screened on 2% low range ultra agarose, followed by 15 cycles of polymerase chain reaction (PCR) amplification using Phusion DNA polymerase. After quantification using a Qubit 4.0, RNA‐Seq libraries were sequenced using a NovaSeq 6000 sequencer (2 × 150 bp read length).

### 2.7 Data analysis

To identify differentially expressed genes (DEGs) between samples, the expression level of each transcript was calculated using the transcripts per million reads method. Genes with a fold change >1.5 and *p* < .05 were considered differentially expressed. GO and KEGG pathway analyses of functionally annotated gene clusters were performed using the OmicStudio tools (https://www.omicstudio.cn/tool) for those DEGs up‐ or downregulated in CUMS rats reversed by EA treatment (J. Huang et al., 2021). Protein–protein interaction (PPI) network analysis was performed using the STRING online software (http://string‐db.org/) for those DEGs up‐ or downregulated in CUMS rats reversed by EA treatment.

### 2.8 Validation experiments with real‐time quantitative PCR (qRT‐PCR)

To confirm the reliability of the RNA‐Seq data, we selected four DEGs for qRT‐PCR analysis to determine their relative expression in the hippocampus. Total RNA was extracted with a SPARKeasy Tissue/Cell RNA Rapid Extraction Kit (AC0202, Shandong Sikejie Biotechnology Co., Ltd). Then, 4 µg of isolated total RNA was transcribed to cDNA through the SPARKscript II RT Plus Kit (AG0304, Shandong Sikejie Biotechnology Co., Ltd.) with oligo‐dT primers. The reaction was conducted in Mastercycler Personal Thermal Cyclers (Eppendorf) with the following thermal profiles: 42°C for 2 min; 50°C for 15 min; 85°C for 5 min; and 4°C indefinitely. Then, qRT‐PCR was performed using 2× SYBR Green qPCR Mix (AH0104, Shandong Sikejie Biotechnology Co., Ltd) in a Biosystems QuantStudio 5 Real‐Time PCR System (Applied Biosystems). The thermal cycles for all analyzed genes were 94°C for 2 min, followed by 40 cycles of 94°C for 10 s and 60°C for 34 s. All primers for selected candidate genes for qRT‐PCR were designed by Beijing Qingke Biotechnology Co., Ltd. Primer sequences are shown in Table 2. The GAPDH gene was used as an internal control to normalize gene expression. The 2^−△△Ct^ method was used to calculate the relative expression levels.

**TABLE 2:** Primer sequences of the selected genes.

Gene	Sequence (5′–3′)
Col1a2	Upstream: TCAGGGTGTTCAAGGTGGCAAAG Downstream: GACCAGCAGGACCAGGGAGAC
Col3a1	Upstream: CCAGGTGGACCAGGCAATGATG Downstream: AGGACCAGGGCGACCACTTTC
Pamb9	Upstream: GTGTCGTGGTGGGCTCTGATTC Downstream: GTCCGCTATGGCTTGGGCATC
Tap1	Upstream: TTTGCCAACGAGGAGGGAGAGG Downstream: CTTCAGCAGCATTCCCGAGACAC

### 2.9 Statistical analysis

Statistical analysis was performed using GraphPad Prism 7. All data are presented as the means ± standard deviations (SD). The Shapiro–Wilk test was applied first to verify the normality of the distribution. When the distribution was close to normal, the data were analyzed by one‐way analysis of variance with the least significant difference post hoc test. When the data were not normally distributed, a nonparametric Kruskal–Wallis test was used. Statistical significance was set at *p* < .05.

## 3 RESULTS

### 3.1 EA ameliorated CUMS‐induced depressive‐like behaviors

The body weights of the rats in the CUMS, EA, and Sham EA groups were significantly lower than those in the Con group (Figure 1b). Rats subjected to CUMS and Sham EA exhibited a marked decrease in sucrose consumption and time spent in the central area and a prolonged immobility time, which were not observed in rats subjected to CUMS and treated with EA (Figure 1c,d).

### 3.2 Regulation of transcripts in the hippocampus

**FIGURE 2:**
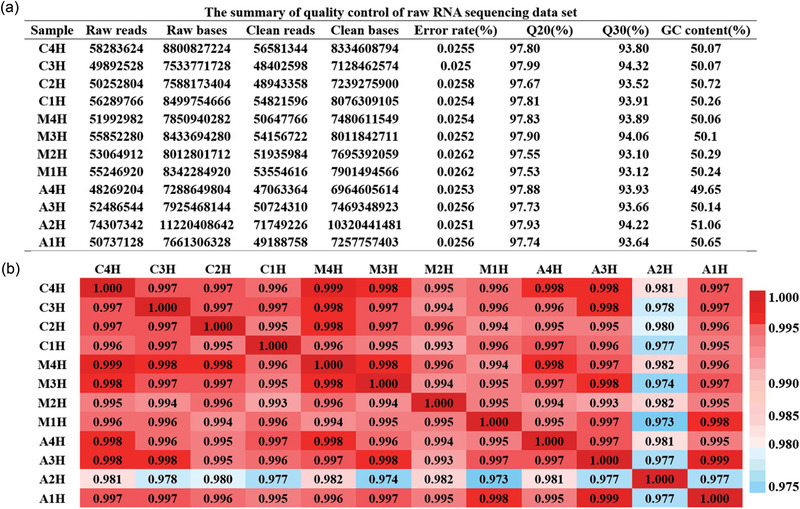
Quality control of the raw RNA sequencing dataset of the hippocampus. (a) Summary of the quality control of the raw RNA sequencing dataset showing the raw reads, clean reads, error ratios, and Q20 and Q30 values of the 12 samples. (b) Heatmap of the correlations between each sample using the Pearson test.

Figure 2a shows the quality control of the sequencing data for each sample. A total of 93.88 GB of clean data were obtained, with each sample having more than 6.96 GB of clean data and a Q30 base percentage of more than 93.1%. We then calculated the correlation value between the two samples based on normalized expression results and generated a correlation heatmap (Figure 2b).

**FIGURE 3:**
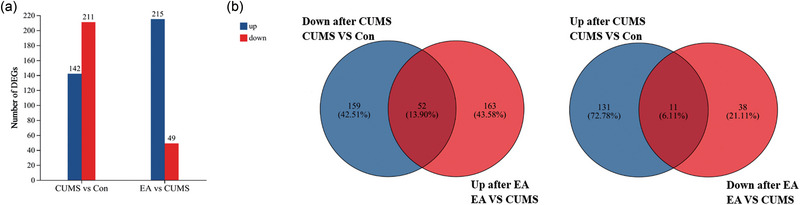
Regulation of transcripts in the hippocampus of control group (Con group) rats, chronic unpredictable mild stress (CUMS) group rats, and electroacupuncture (EA) group rats. (a) Histogram showing the statistics of up‐ and downregulated differentially expressed genes (DEGs). (b) Venn diagram showing the number of unique and shared DEGs with a fold change >1.5 and a *p*‐value < .05.

Our data revealed 727 DEGs with cutoffs of fold changes >1.5 and *p* < .05. A histogram of the up‐ and downregulated genes is shown in Figure 3a. Compared to those in control rats, 353 genes were significantly differentially expressed in rats subjected to CUMS, of which 142 were upregulated and 211 were downregulated. Compared with rats subjected to CUMS, EA rats had 264 DEGs, of which 215 were upregulated and 49 were downregulated. In addition, Venn analysis was used to determine the possible markers associated with the antidepressant effect of EA. We found that the changes in the expression of a total of 63 genes in rats subjected to CUMS were reversed by EA treatment, including 52 of the 211 downregulated genes and 11 of the 142 upregulated genes (Figure 3b). In addition to the 63 differential genes described above, EA did not reverse or exacerbate the expression of other differential genes in rats subjected to CUMS. The results indicate that the mechanisms underlying the effects of EA on depression are related to the regulation of multiple genes.

### 3.3 GO annotation analysis of the DEGs

**FIGURE 4:**
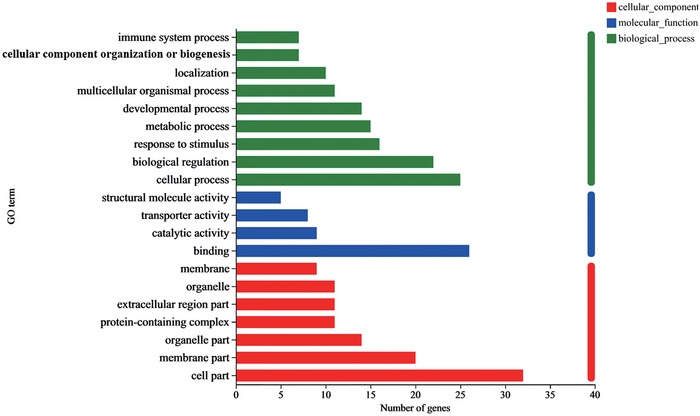
Go analysis of differentially expressed genes (DEGs). GO analysis showing the top 20 significantly enriched DEGs for biological processes, cellular components, and molecular functions of DEGs. GO terms are plotted as the ordinate, and the gene number is plotted as the abscissa.

To better understand the specific functions of DEGs in the hippocampus of CUMS rats with EA, we performed GO analysis of the biological processes, cellular components, and molecular functions of DEGs (Figure 4). Genes were differentially downregulated or upregulated in the CUMS group, but these changes tended to be reversed after EA treatment; their biological processes were enriched in cellular process, biological regulation, response to stimulus, and immune system process, suggesting that strong immune responses occurred after EA treatment in the rats subjected to CUMS. The cellular components mainly included cell part, membrane part, organelle part, protein‐containing complex, and extracellular region part, indicating that multiple membrane components were involved in CUMS‐exposed rats receiving EA. The molecular function terms were focused on binding and catalytic activity.

Taken together, the GO analysis indicated that immune responses are key physiological processes in the EA treatment of rats subjected to CUMS.

### 3.4 KEGG annotation analysis of the DEGs

**FIGURE 5:**
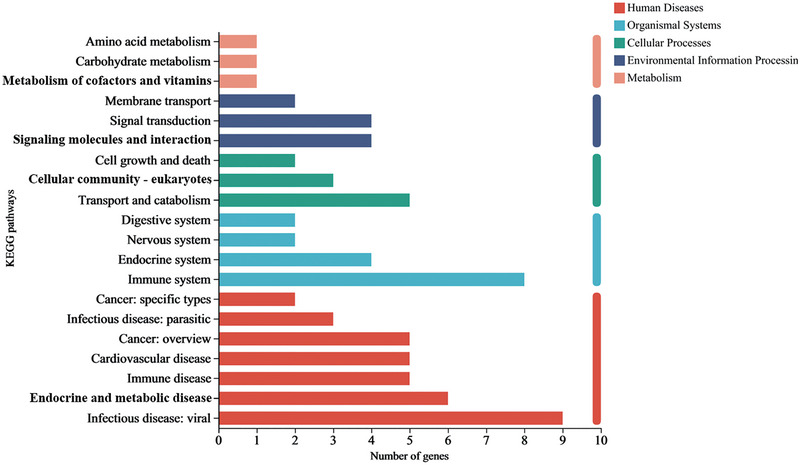
KEGG pathway classification of differentially expressed genes (DEGs). The top 20 significantly enriched KEGG pathways are shown. The KEGG classifications are plotted as the ordinate and the number of DEGs is plotted as the abscissa.

To determine the putative functional biochemical pathways of the predicted proteins encoded by the DEGs, KEGG annotation analysis was performed on the DEGs (Figure 5). Among the 20 pathways, the DEGs were enriched in the infectious disease: viral, endocrine and metabolic disease, immune disease, immune system, endocrine system, nervous system, transport and catabolism, cellular community—eukaryotes, signal transduction, and signaling molecules and interaction pathways, which indicated that EA mainly improves depression‐like behaviors in rats subjected to CUMS by regulating the immune system function.

### 3.5 PPI network analysis of DEGs

**FIGURE 6:**
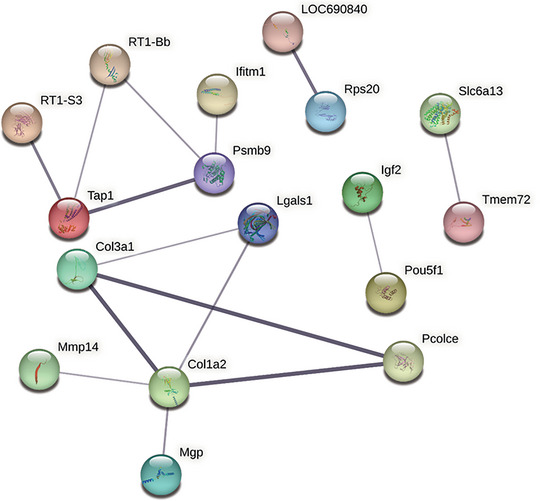
Protein–protein interaction (PPI) network analysis of differentially expressed genes (DEGs). STRING analysis of the PPI networks of DEGs whose expression changes could be reversed by electroacupuncture (EA) treatment in rats subjected to chronic unpredictable mild stress (CUMS). Network nodes represent proteins. Edges represent protein‒protein associations, which include known interactions, predicted interactions, text mining, coexpression, and protein homology.

A PPI network of DEGs was constructed using the STRING online tool. The CytoHubba plugin of Cytoscape software was used to identify the top 10 genes by using Degree and MCC; then the intersection was used to obtain the hub genes. The following genes were identified: Colla2, Col3a1, Psmb9, Tap1, Pcolce, RT1‐Bb, Lgals1, RT1‐S3, LOC69084, and Rps20 (Figure 6), which are involved mainly in the regulation of immune system function. These 10 genes were all downregulated after rats were exposed to CUMS and then reversed after EA treatment. In this interaction network, the top three genes were Colla2, Col3a1, Psmb9, and Tap1.

### 3.6 qRT‐PCR validation results

**FIGURE 7:**
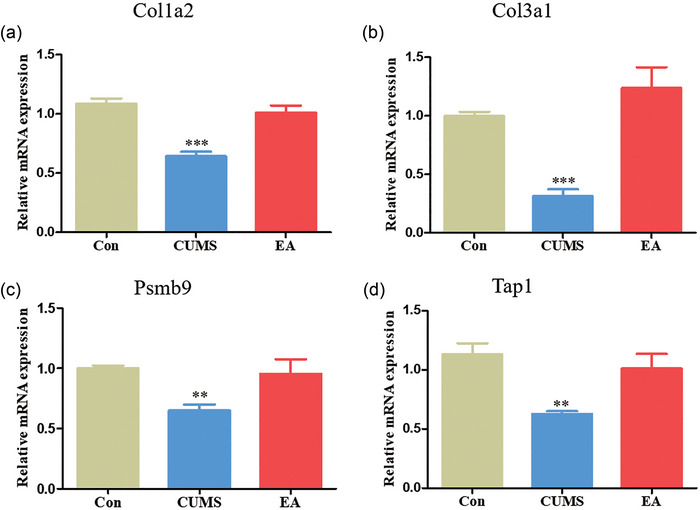
Real‐time quantitative polymerase chain reaction (qRT‐PCR) analysis of four key differentially expressed genes. Data are expressed as the means ± SD; ^**^ *p *< .01, ^***^ *p *< .001, compared with the control group (Con group).

To confirm the RNA‐Seq data, a total of four DEGs, which were among the top three genes, were selected for qRT‐PCR validation. The results are shown in Figure 7. Colla2, Col3a1, Psmb9, and Tap1 were all downregulated in the hippocampus of rats subjected to CUMS. After EA treatment, the downregulation of all these genes was reversed. All the results of fluorescence quantitative analysis outlined above were consistent with the transcriptome sequencing results, indicating that our transcriptome sequencing data were accurate and reliable and could be used as reference data for future studies. It can be concluded that the key DEGs detected in this study are significant for studying the mechanisms by which EA at GV20 and LR3 improves depression.

## 4 DISCUSSION

Acupuncture points are specialized meridian patterns rich in nerve, vascular, and immune cells that are associated with specific organs and regulate the related body functions (Song et al., 2019). According to the theory of acupuncture, the selection of different acupoints has a great influence on the therapeutic effects of acupuncture (Yang et al., 2020). The Governor Vessel is the most commonly used meridian for people with depression, as it is the only one of the 14 meridians that directly connects the head and brain (Yang et al., 2022). At this meridian, GV20 and *Yin‐tang* (GV29) are frequently used points to promote recovery from depression. In addition, Liver Qi stagnation is also a cause of depression and can lead to symptoms such as mental stress, low mood, decreased activity, and low self‐esteem (Wen et al., 2018). The liver meridian acupoints *Xingjian* (LR2), LR3, and *Qimen* (LR14) are used in standard acupuncture protocols to regulate Liver Qi movement. In summary, the application of meridians and acupoints for the treatment of depression focuses on head acupoints (such as GV20 and GV29) and limb acupoints (such as LR3 and LR2). This approach makes good use of superior–inferior acupoint matching. Our previous study showed that GV20 and LR3 are effective acupoints for treating depression (Y. Wang et al., 2024). Therefore, GV20 and LR3 were selected to explore the effects and mechanisms of EA on depression.

The extracellular matrix refers to the entire extracellular environment surrounding a cell and is composed of various complex macromolecular substances. Collagen proteins, such as those encoded by the Col1a2 and Col3a1 genes, are important components of the extracellular matrix. The Col1a2 gene encodes type I collagen, which is abundant in multiple tissues in the body and supports body shape and function. Recent studies have revealed that Col1a2 plays a key role in depression. Miao et al. (2019) reported that the Col1a2 gene, which is involved in the neurotrophin signaling pathway, is closely related to depression in Alzheimer's disease patients. There is a close correlation between depressive/anxiety disorders and glioblastoma multiforme. LTBP1 could be a potential bridge linking the two diseases through the regulation of the extracellular matrix. Analysis of PPI and hub genes revealed that collagen‐related genes such as Col1a2 could be the most crucial molecules that are differentially expressed with LTBP1 (Fu et al., 2020). Heart failure comorbid with depression may be associated with five hub genes, including Stat4, Cd83, Cx3cr1, Col1a2, and Sh2d1b. According to the validated datasets, Stat4 and Col1a2 were particularly strongly associated with the comorbidity of heart failure and depression. Our research results suggested that the expression of the Col1a2 gene was downregulated in rats subjected to CUMS, and this change could be reversed after EA at GV20 and LR3.

The Col3a1 gene encodes the type III collagen α‐1 chain, and different variations in this gene affect the recurrence and prognosis of stroke and also play important roles in cortical development. The biallelic mutation of Col3a1 can lead to a special brain phenotype, including bilateral frontoparietal polymicrogyria with cobblestone variants, cerebellar microcysts, and white matter abnormalities (Horn et al., 2017). In this study, we found that Col3a1 expression was downregulated in depressed individuals but returned to normal levels after EA at GV20 and LR3. This warrants further study.

Depression is also thought to be caused by an imbalance in the peripheral or central immune response (Beurel et al., 2020; K. Huang et al., 2022). The innate immune system is the first line of defense against infection and stressors. Most relevant from a pathophysiological perspective of depression is the immune system's ability to recruit immune cells through the production of cytokines, activation of the complement cascade, and subsequent activation of the adaptive immune system through antigen presentation. Genes involved in antigen processing and presentation, such as transporter 1 (Tap1), which is involved in antigen processing, and proteasome subunit‐β type‐9 (Psmb9), have been shown to play potential roles in a variety of immune diseases. The proteasome is a fundamental complex that helps regulate T‐cell function (Minelli et al., 2015). Psmb9 encodes the catalytic subunit of the immunoproteasome, a modified proteasome that processes class I peptides of the major histocompatibility complex (MHC‐I) and viral proteins (Vriend et al., 2023). It has been shown that the proteasome genes Psmb7, Psmb9 (one of the hub genes in our study), and Psmb13 may play a role in susceptibility to antidepressant responses. In addition, Psmb9 rs1043307 was positively correlated with anxiety disorders in patients with major depressive disorder comorbidities, although this result was not significant after adjusting for multiple comparisons (Minelli et al., 2015). Recent bioinformatic analysis demonstrated that immune regulatory mechanisms mediated by key genes (for example, Psmb2, Psmb6, Psmb8, and Psmb9) may play a critical role in abnormalities in the aberrant fetal gene expression profiles that lead to severe prenatal depression (Liu et al., 2019). Psmb9 reached a maximum level 24 h after IFN‐α and poly(I:C) exposure, which was before the peak levels of the above cytokines and chemokines were observed, which might be useful markers of imminent depression (Hoyo‐Becerra et al., 2015).

The Tap gene plays an important role in intracellular peptide antigen presentation. Tap is a heterodimeric complex composed of Tap1 and Tap2 that utilizes ATP to transport cytosolic peptides across membranes to the endoplasmic reticulum. In the endoplasmic reticulum, Tap forms peptide‐loading complexes with other components that load high‐affinity peptides onto nascent MHC‐I molecules, which are then transported to the cell surface and presented to CD8^+^ T cells. Tap also plays a crucial role in the transport of peptides to phagosomes and endosomes during cross‐presentation by dendritic cells. Because Tap plays a key role in classical MHC‐I presentation and cross‐presentation, its expression and function are often impaired to avoid recognition by cytotoxic CD8^+^ T cells (Embgenbroich & Burgdorf, 2018). We demonstrated that EA at GV20 and LR3 reversed the decrease in Tap1 expression in the hippocampus of rats subjected to CUMS, indicating that EA can ameliorate immune function defects in rats subjected to CUMS.

Immune dysregulation and depression are associated or correlated with each other (Beurel et al., 2020; Drevets et al., 2022). Mammals are protected by the immune system from infectious agents and many types of insults that cause injury. The innate immune system recruits immune cells through the production of cytokines, activation of the complement cascade, and subsequent activation of the adaptive immune system through antigen presentation (H. Wang et al., 2022). Adaptive immunity involves immune memory, which is a highly specific defense mechanism in which the body responds to specific pathogens (Beurel et al., 2020). Immune imbalance can result in impaired hippocampal structure and function and possibly even cell death (Yang et al., 2022). The biological processes of genes that were differentially downregulated or upregulated in the CUMS group but tended to be reversed after EA treatment were enriched in biological regulation, response to stimulation and immune system processes. These results suggested a strong immune response in rats subjected to CUMS. These genetic changes may underlie the manifestation of depression in this model (Zang et al., 2023). The mechanism by which EA improves depression is closely related to the regulation of multiple genes involved in immune responses (Y. Wang et al., 2017). This study provides a useful reference for the study of the central mechanism of EA in the treatment of depression, provides experimental evidence for the observed clinical effects of EA in the treatment of depression, and provides guidance for the comprehensive treatment of depression.

This study has some obvious limitations. For example, we used only male rats and did not address sex‐related differences, which limits the generalizability of our results. It is worth exploring whether the DEGs we identified above exhibit sex differences in depression. In addition, we observed that EA at GV20 and LR3 attenuated CUMS‐induced depression‐like behaviors by regulating the expression of specific genes, such as Colla2, Col3a1, Psmb9, and Tap1. However, the mechanisms involved are broad and complex, and we did not delve into the interrelationships between these DEGs and depressive‐like behaviors. Future research should further identify the upstream targets involved. Furthermore, whether the knowledge acquired in rodents can be extended to humans is a key question. There are many challenges in breaking down the barriers between preclinical animal studies and clinical investigators studying human subjects. However, cross‐species comparisons must be performed to test this hypothesis.

## 5 CONCLUSION

In brief, our study suggested that EA treatment at the GV20 and LR3 acupoints may alleviate depression induced by CUMS. In addition, our bioinformatics analysis comprehensively revealed the mechanisms by which EA treats depression. Our data will contribute to future studies aimed at gaining insight into the role of these genes in depression.

## AUTHOR CONTRIBUTIONS

Xiaoli Chang contributed to the study design, data collection, and data analysis; wrote the first draft; and revised the manuscript. Ying Wang, Yi Hou, and Weilu Cheng contributed to the data collection. Shaozong Chen contributed to the study design, data analysis, and manuscript revision. All authors reviewed and accepted the content of the final manuscript.

## CONFLICT OF INTEREST STATEMENT

The authors declare no conflicts of interest.

### PEER REVIEW

The peer review history for this article is available at https://publons.com/publon/10.1002/brb3.70045.

## Data Availability

The data generated in this study can be found in the GenBank SRA via BioProject PRJNA1121507.
